# Icariin improves brain function decline in aging rats by enhancing neuronal autophagy through the AMPK/mTOR/ULK1 pathway

**DOI:** 10.1080/13880209.2021.1878238

**Published:** 2021-02-08

**Authors:** Jie Zheng, Shanshan Hu, Jinxin Wang, Xulan Zhang, Ding Yuan, Changcheng Zhang, Chaoqi Liu, Ting Wang, Zhiyong Zhou

**Affiliations:** a Medical College of China Three Gorges University, Yichang, P. R. Chinas; b Department of Pharmacy, College of Medicine, New Drug Innovation and Development Institute, Wuhan University of Science and Technology, Wuhan, Hubei Province, China; c Hubei Province Key Laboratory of Occupational Hazard Identification and Control, Wuhan University of Science and Technology, Wuhan, Hubei Province, China

**Keywords:** Senescence, macroautophagy, neurodegenerative diseases, *Epimedium brevicornu* Maxim

## Abstract

**Context:**

Icariin (ICA) is the main active ingredient of *Epimedium brevicornu* Maxim (Berberidaceae), which is used in the immune, reproductive, neuroendocrine systems, and anti-aging.

**Objective:**

To evaluate the effect of ICA on natural aging rat.

**Materials and methods:**

16-month-old Sprague–Dawley (SD) rats were randomly divided into aging, low and high-dose ICA groups (*n* = 8); 6-month-old rats were taken as the adult control (*n* = 8). Rats were fed regular feed (aging and adult control) or feed containing ICA (ICA 2 and 6 mg/kg group) for 4 months. HE and Nissl staining were used to assess pathological changes. Western blot was used to test the expression of autophagy (LC3B, p62, Atg5, Beclin1) and p-AMPK, p-mTOR and p-ULK1 (ser 757). Immunofluorescence was used to detect the co-localization of LC3 and neurons.

**Results:**

ICA improved neuronal degeneration associated with aging and increased the staining of Nissl bodies. Western blot showed that ICA up-regulated autophagy-related proteins LC3B (595%), Beclin1 (73.5%), p-AMPK (464%) protein (*p* < 0.05 vs. 20 M) in the cortex and hippocampus of aging rats, down-regulated the expression of p62 (56.9%), p-mTOR (53%) and p-ULK1 (ser 757) (65.4%) protein (*p* < 0.05 vs. 20 M). Immunofluorescence showed that the fluorescence intensity of LC3 decreased in the aging rat brain, but increased and mainly co-localized with neurons after ICA intervention.

**Conclusions:**

Further research needs to verify the expression changes of AMPK/mTOR/ULK1 and the improvement effect of ICA in elderly. These results will further accelerate the applications of ICA and the treatment for senescence.

## Introduction

According to the data of Chinese National Bureau of Statistics, in 2018, the aging population in China has reached 249 million, which approximately equivalent to 17.9% of the total population (Zhang et al. 2019). With the acceleration of the social aging process, aging associated chronic diseases including neurodegeneration, cancer, cardiovascular disease, metabolic disease and macular degeneration will bring heavy medical and economic burden (Pattison and Korolchuk 2018). Among them, neurodegenerative diseases are considered to be an important factor affecting the quality of life (Erickson et al. 2012; Bertoldi et al. 2017; Currais et al. 2017; Poulose et al. 2017). Globally, almost 46 million people suffer from neurodegenerative diseases and it is estimated that these diseases have caused more than an $818 billion economic burden (Paillusson et al. 2016). Therefore, delaying or even improving the progression of neurodegenerative diseases in elderly is an urgent problem.

Macroautophagy (hereafter, autophagy) is a highly conserved catabolic process that transports damaged cytoplasmic components to lysosomes for degradation and reuse (Mendonca et al. 2017). A large amount of evidence confirms that autophagy is decreased during aging (Simonsen et al. 2008; Ling and Salvaterra 2011; He et al. 2013; Chang and Hansen 2018; Anuradha et al. 2019). The reduction of autophagy is closely related to many neurodegenerative diseases, including Parkinson's disease, Alzheimer's disease, Huntington's disease, and amyotrophic lateral sclerosis (Nixon 2013; Damme et al. 2015; Wong and Holzbaur 2015; Dikic and Elazar 2018). In addition, enhance autophagy through pharmacological methods can effectively improve the potential of neurons to resist stimuli and ameliorate cognitive function. For instance, Sphingosine kinase 2 activates autophagy through promoting the dissociation of Beclin1 with Bcl-2, which reduce the damage of neurons caused by glucose oxygen deprivation (Song et al. 2017). Caloric restriction, known as an effective strategy to activate autophagy, improves learning and memory function in aged mice (Dong et al. 2015). These data indicate that autophagy is an effective target to improve neurodegenerative diseases during aging.

AMP-activated protein kinase (AMPK), an energy regulator of eukaryotic cells, also plays an important role in regulating autophagy. On the one hand, AMPK can promote autophagy by inhibiting the activation of mTORC1 through phosphorylating tuberous sclerosis complex 2 (TSC2) and Raptor. On the other hand, AMPK can directly phosphorylate multiple sites in the autophagy initiating factor Uncoordinated-51 like kinase 1 (ULK1), such as S317, S467, S555, T575, S637 and S777 etc., activate autophagy (Zang et al. 2006; Gwinn et al. 2008; Kalender et al. 2010; Wong et al. 2013). AMPKα1 deficiency impairs autophagy which leads to monocyte differentiation and decrease monocyte/macro-phage survival (Zhang et al. 2017). Furthermore, increasing p-AMPK can activate autophagy and rescue SHSY-5Y cells degeneration in PD model (Gong et al. 2016). Previous studies showed that the activation of AMPK was significantly decreased during aging (Cai et al. 2020; Lin et al. 2020). This evidence confirms that AMPK mediated autophagy is thought to be an important target to maintain neurons homeostasis during aging.

Icariin (ICA), a flavonoid, is the main active ingredient of *Epimedium brevicornu* Maxim (Berberidaceae), a traditional Chinese herbal medicine which is widely used for its pharmacological effects of the immune system, reproductive system, neuroendocrine systems, anti-aging, etc. (Chen et al. 2019; Zhou et al. 2019; Xu et al. 2020). It has been well documented that ICA has a good neuroprotective effect. In the traumatic brain injury model, ICA can improve the cognitive dysfunction through increased cholinergic function of the hippocampus (Zhang et al. 2018). In addition, ICA can attenuate glial cells-mediated neuroinflammation and exert dopamine neuroprotection via an Nrf2-dependent manner (Zhang et al. 2019). However, whether ICA can improve the level of neuronal autophagy during aging and the underlying mechanism have not been elucidated. In the present study, we evaluated the effect of ICA on the neuron’s autophagy level in the cortex and hippocampus using naturally aging rat model, and further investigated the underlying mechanism through the AMPK/mTOR/ULK1 pathway.

## Materials and methods

### Animals and treatment

Sprague-Dawley (SD) rats were provided and fed by Animal Centre of China Three Gorges University, Yichang, China. Animals were housed in an environmentally controlled feeding room (free access to food and water, 23 ± 3 °C, 12 h light/dark cycle). This study was approved by the China Three Gorges University Council on Animal Care Committee. The handling, experimental procedures, and care of the animals were carried out in accordance with the National Institutes of Health Guide for the Care and Use of Laboratory Animals.

Animals were randomly divided into four groups and treated with various regimens: (a) adult control group (*n* = 8, normal feed, 2-month old rats were purchased and raised to 6-month old); (b) aging control group (*n* = 8, normal feed, 16-month old rats were purchased and raised to 20-month old); (c) ICA 2 mg/kg treated group (*n* = 8, contains ICA feed, 16-month old rats were purchased and food administration for consecutive 4 months until they were 20-month old); (d) ICA 6 mg/kg treated group (*n* = 8, contains ICA feed, 16-month old rats were purchased and food administration for consecutive 4 months until they were 20-month old). Briefly, we measured the daily food intake of each rat, and calculated the ICA content per kilogram of feed based on the average weight and food intake. Rat feed containing ICA is processed by Beijing Huafukang Biotechnology Co., Ltd. At a predetermined time, rats were anaesthetized with intraperitoneal injecting urethanes. The cortex and hippocampus were snap frozen for further experiments.

### Antibodies and reagents

Icariin (ICA, purity >98.61%) was purchased from Cheng Du Purechem-Standard Co., Ltd. (Chengdu, China). Haematoxylin, eosin, Nissl staining solution and ECL chemiluminescence detection kit were purchased from Servicebio (Wuhan, China), RIPA buffer and BCA protein assay kit were purchased from Applygen (Beijing, China), PVDF was purchased from Millipore (MA, USA). Anti-LC3B (#83506), anti-p-ULK1 (ser757) (14202S), anti-p-mTOR (#5536), anti-p-AMPK (#2535) were purchased from Cell Signalling Technology (MA, USA). Anti-LC3 (14600-1-AP) was purchased from Proteintech (Wuhan, China). Anti-p62 (ab56416) and anti-Beclin1 were purchased from Abcam (Cambridge, UK). Anti-Atg5 (NB110-53818SS) was purchased from Novus Biologicals (CO, USA). Anti-NeuN (#2967854) was purchased from Millipore (MA, USA). Alexa Fluor 488 Donkey anti-Mouse IgG (H + L) (138499) and Alexa Fluor 594 Donkey anti-Rabbit IgG (H + L) (140019) were purchased from Jackson ImmunoResearch (PA, USA).

### HE staining

The perfused brain tissues were soaked in 4% paraformaldehyde at least 24 h, dehydrated in an ascending ethanol series, and equilibrated with xylene. Then, embedded in paraffin and cut for 5 μm thick sections. After conventional dewaxing and hydration, sections were stained with haematoxylin and eosin. The pathomorphological changes in cortex and hippocampus were observed by microscope according to double-blind method.

### Nissl staining

After dewaxed with xylene and gradient alcohol to water, slices were incubated with Nissl staining solution for 10 min at 37 °C, then washed in double distilled water quickly, hydrated in 95% ethanol for 10 sec. Finally, samples were sealed with neutral gum and observed under microscope according to double-blind method.

### Immunofluorescence

After melting paraffin at 60 °C for 4 h, the brain sections were deparaffinized and rehydrated conventionally. Sections were heated with citric acid buffer using microwave for 20 min, then incubated with 3% H_2_O_2_ for 10 min at room temperature (RT). After blocking with 5% BSA for 1 h at RT, the tissue sections were incubated with primary antibody (anti-LC3 and anti-NeuN) diluted in 1% BSA and 0.5% Triton X-100 overnight at 4 °C. Followed by rewarming for 30 min at RT, washing with PBS. Corresponding Alexa Fluor antibody with fluorescence were incubated for 1 h avoiding light at RT. After washing, the tissue sections were stained with DAPI at 25 °C for 10 min. Finally, adding anti-fluorescence quencher agent on the sections and sealing. Image acquisition under confocal microscope (Nikon, A1R+).

### Western blot

Cortical and hippocampal tissues (20 mg) were lysed in RIPA lysis buffer. BCA protein assay kit determine the protein concentration. Equal amount of protein (30 μg) was loaded onto 8–15% SDS-PAGE gal, and transferred to PVDF membranes. After blocking in 5% skim milk in TBS-T buffer at room temperature for 1 h, the membranes were followed by incubation with primary antibody at 4 °C overnight. After being washed in TBS-T buffer, the membranes were incubated with secondary antibodies at room temperature for 1 h. The results of protein bands were analysis by ImageJ software.

### Statistical analysis

The date were expressed as mean ± SD. Results were analyzed by one-way ANOVA of variance followed by Tukey's *post hoc* test, and *p* < 0.05 was considered to be statistically significant.

## Results

### ICA effectively alleviates the morphological changes of cortex and hippocampus in aging rats

**Figure 1.**
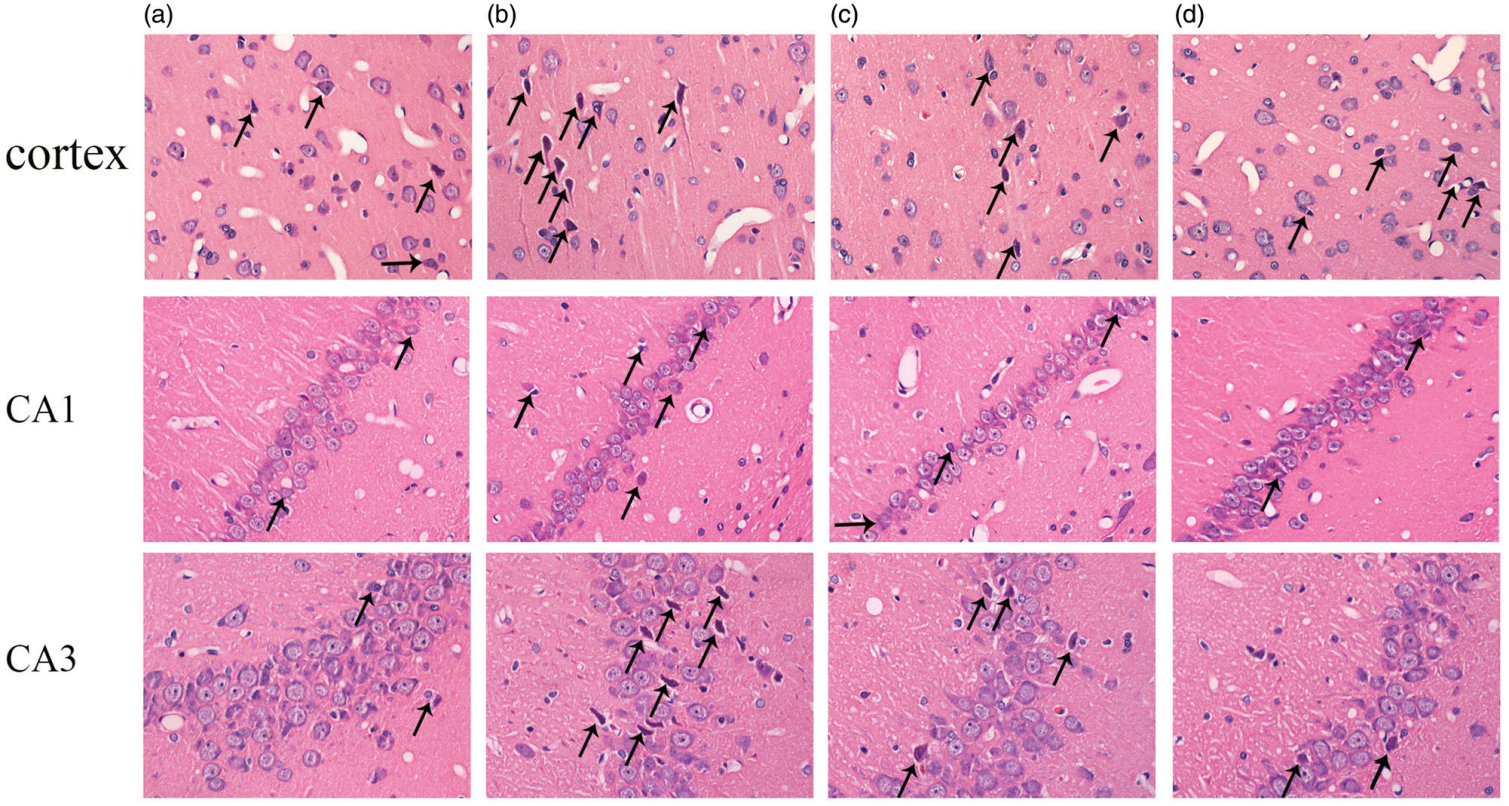
Protective effects of ICA against aging associated neuron damage in the cortex and hippocampus CA1 and CA3 regions of rats. After HE staining, the morphological changes of rat cortex and hippocampus was observed under an optical microscope (400×). In the cortex and CA3 region, the neurons of 6 M are normal in shape, and degeneration is rare; the neurons of 20 M have no obvious changes in density, but the degeneration neurons increased significantly. In the CA1 region, neurons are arranged tightly in adult rat, the number of cell layers is 2–3. However, the neuronal number of aging rats is reduced and the number of cell layers decreased to 1–2 layers. The arrows indicated damaged neurons.

As shown in Figure 1, HE staining was used to evaluate the morphological changes of cortex and hippocampus in aging rats. In the cortex, compared with adult group, neurons are extensively damaged in the aging rat (20 months old) with the specific performances as: unclear membrane boundaries, nuclear psychosis, decreased cytoplasm and dyeing depth. In the hippocampus, besides the similar changes to the cortex, we also found that disordered arrangement of neurons of CA3 region and the decreased number of neuronal layers in the hippocampal CA1 region in the aging rats. After ICA intervention, both of the above morphological changes can be alleviated effectively.

### ICA effectively improves the condition of neurons in aging rats

**Figure 2.**
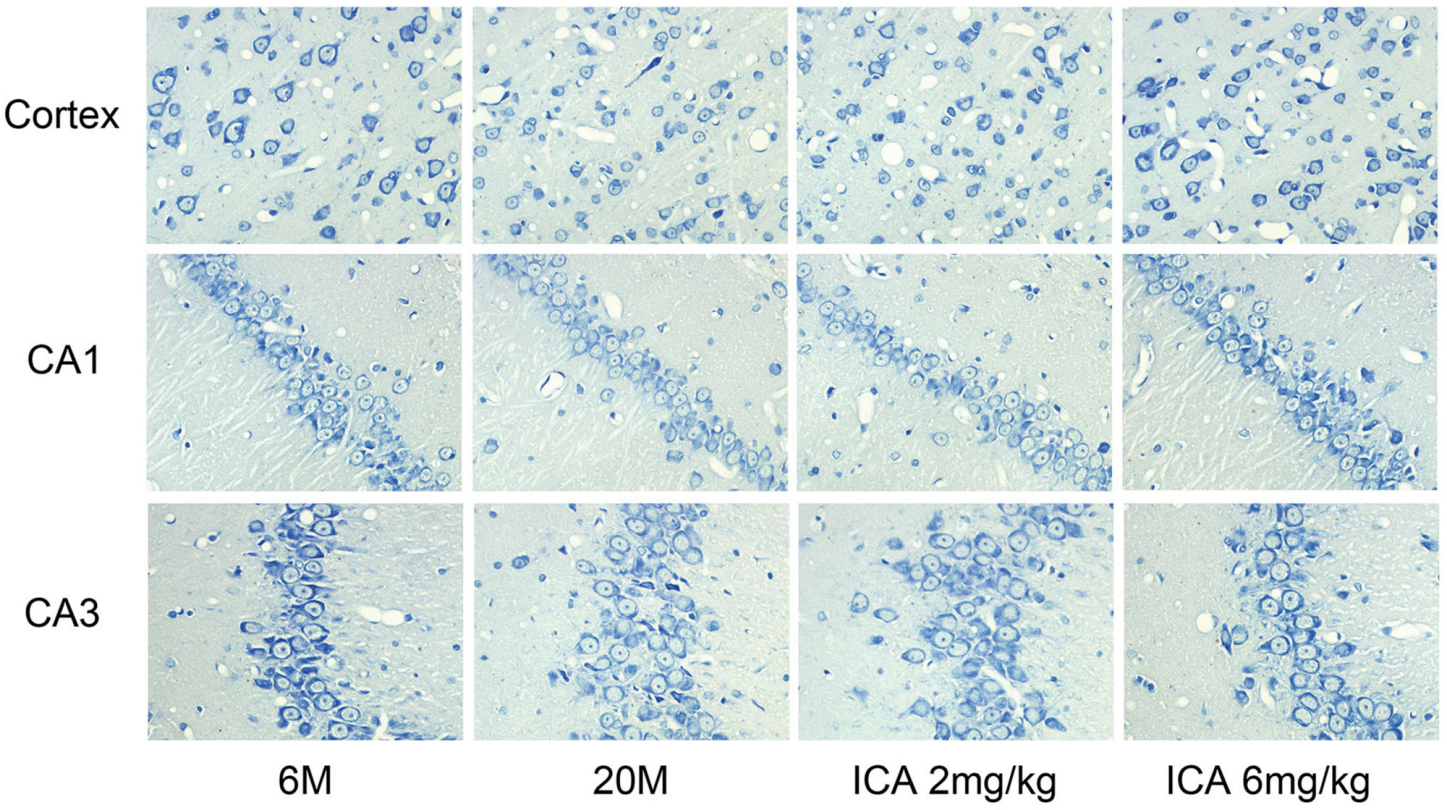
ICA effectively improves the condition of neurons (400×). Nissl body is mainly distributed in the cytoplasm of neurons except axons, and its main function is to synthesize protein. Compared with 6 M, the staining of Nissl bodies in cortex and hippocampus of 20 M rat became lighter, which suggesting the neuronal function is impaired. ICA significantly reversed the trend in a dose-dependent manner.

Nissl body is a characteristic structure of neurons, and its degree of staining is a crucial indicator for evaluating the number and function of neurons. As shown in Figure 2, compared with the adult group, the Nissl staining of aging rat was significantly lighter both in cortex and hippocampal neurons, and ICA treatment could effectively reverse this change. These results indicated that ICA could effectively improve the neurons damage during aging.

### ICA effectively improves autophagy level in cortex and hippocampus of aging rats

**Figure 3.**
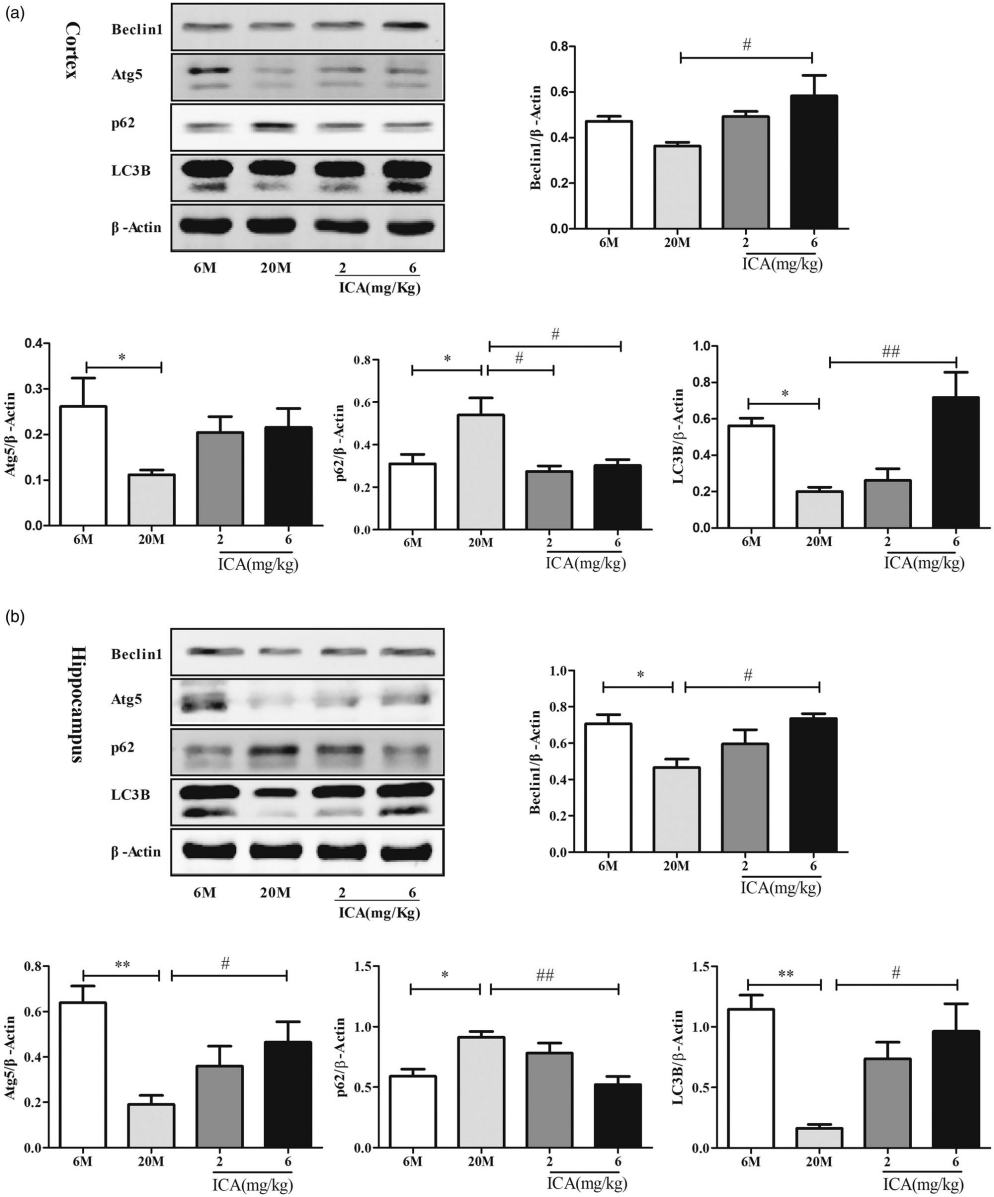
ICA enhances autophagy in the cortex (a) and hippocampus (b) of aging rat. The level of autophagy was significantly reduced in aging rat brain, and ICA intervention can increase autophagy in the cortex and hippocampus. (*n* = 4, *p < 0.05 vs. 6 M; **p < 0.01 vs. 6 M; #*p* < 0.05 vs. 20 M; ##*p* < 0.01 vs. 20 M).

Currently, autophagy was considered to play a critical role in maintaining neuronal homeostasis. To further clarify the impact of ICA on autophagy, autophagy related protein levels (specifically including: Atg5, LC3B, p62, Beclin1) were detected by Western blotting. In cortex, as shown in Figure 3(a), compared to adult group, the level of LC3B and Atg5 in aging group were significantly down-regulated (*p* < 0.05). However, the expression of Beclin 1 has no statistical difference. In addition, the expression of p62 was remarkable increased (*p* < 0.01). After high dose of ICA treatment, the level of LC3B (*p* < 0.01) and Beclin1 (*p* < 0.05) protein was up-regulated remarkably. Meanwhile, the expression of p62 was significantly decreased (*p* < 0.05) both in low and high dose, compared to aging group. In hippocampus, as shown in Figure 3(b), the expression of LC3B, Atg5 and Beclin1 were all significantly decreased and p62 remarkable increased in aging group compared with adult. ICA effectively reverses the decline of LC3B, as well as Atg5 and Beclin1. At the same time, ICA effectively decreased the expression of p62 protein.

### ICA improves autophagy levels in cortex and hippocampus of aging rats through AMPK/mTOR/ULK1 pathway

**Figure 4.**
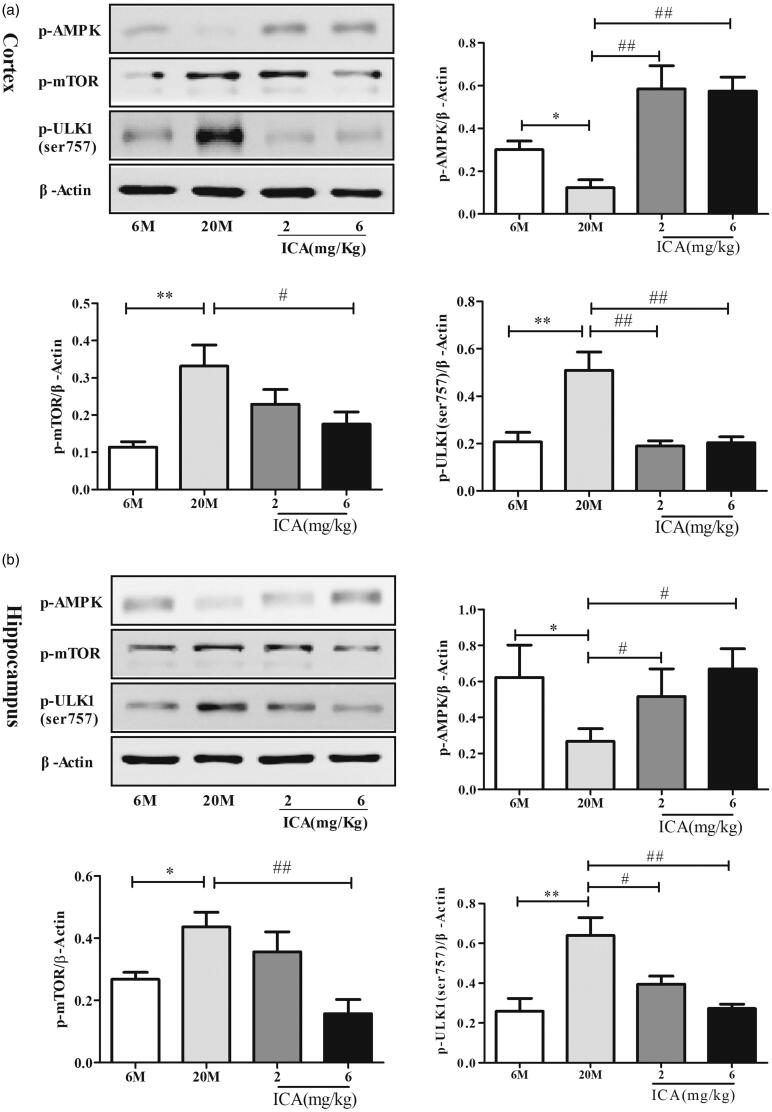
ICA enhances autophagy levels via inducing AMPK-mTOR-ULK1 pathway in cortex (a) and in the hippocampal (b) of aging rats. ICA promotes the phosphorylation of AMPK in the cortex and hippocampus of aging rats, resulting inhibits mTOR phosphorylate and reduces the phosphorylation of ULK1 serine 757. (*n* = 4, *p < 0.05 vs. 6 M; **p < 0.01 vs. 6 M; #*p* < 0.05 vs. 20 M; ##*p* < 0.01 vs. 20 M).

To further elucidate the underlying molecular mechanism mediated autophagy, western blotting was performed in cortex and hippocampus tissue sample to evaluate the expression of p-AMPK, p-mTOR and p-ULK1(ser757). In cortex, p-AMPK level was decreased (*p* < 0.05) in aging group, followed with increased expression of p-mTOR (*p* < 0.01) and ULK1 ser757 (*p* < 0.01). ICA intervention can active AMPK phosphorylation and inhibit mTOR activation obviously which reduce ULK1 (S757) phosphorylation, as shown in Figure 4(a). Hippocampus (Figure 4(b)) showed the same trend with cotex tissue. ICA effectively reversed the decrease of p-AMPK, and down-regulated the expression of p-mTOR and p-ULK1(ser757).

### Co-localization of LC3 and neurons in hippocampus and cortical tissue

**Figure 5.**
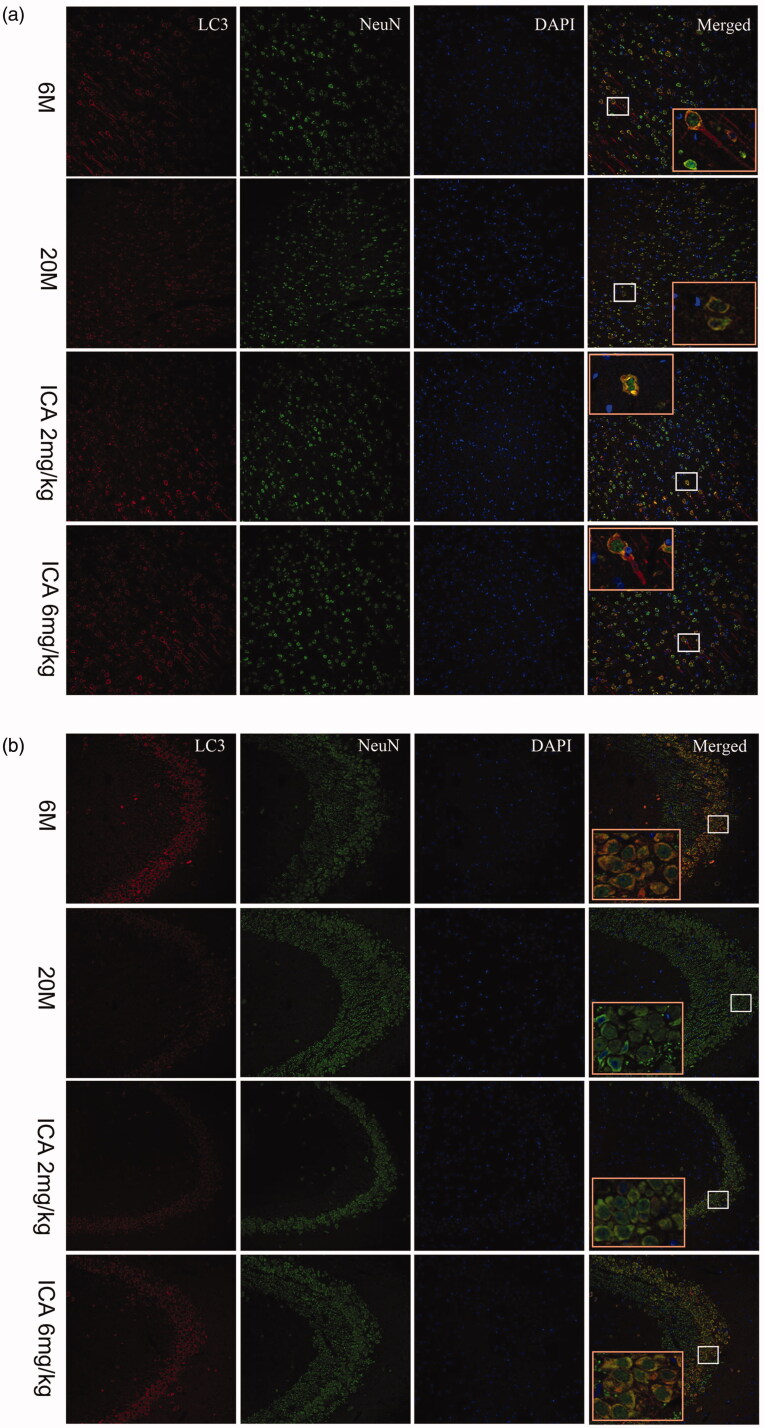
Double-immunofluorescence staining of LC3 and NeuN in cortex (a) and hippocampal CA3 region (b). The expression of LC3 shows in the first column from left; the Neun-labeled neurons shows in the second column; DAPI stains the nucleus and shows in the third column. Compared with 6 M group, the fluorescence intensity of LC3 is significantly reduced in 20 M. After the intervention of ICA, the fluorescence intensity of LC3 increased remarkably. The enlarged image (right) shows the characteristic co-localization image (200×).

As shown in Figure 5, we found that LC3 and NeuN, biomarkers of neurons, were co-localized in cortex and hippocampus. This result indicates that autophagy in the brain mainly occurs in neuronal cells, which plays an important role in maintaining neuronal function homeostasis. It’s remarkable that the fluorescence intensity of LC3 in aging group was lower than that in adult group. After treatment with ICA, more LC3-positive cells were observed in cortex and CA3 region of hippocampus.

## Discussion

Aging-related neurodegenerative diseases are the major cause of disability and death in elderly among the world, with Alzheimer's disease, Parkinson's disease, Huntington and Amyotrophic lateral sclerosis as the common disease (Aguzzi and O'Connor 2010; Stephenson et al. 2018). It is well established that neuronal dysfunction is mainly associated with age-related neuronal degeneration, which has become the primary target of anti-aging drugs (Turlejski and Djavadian 2002). Consistent with previous studies, in this study, we also found significant neuron damage in cortical and hippocampal in aging rat, with the feature of increased neuronal degeneration, nuclear pyknosis in CA3 region and decreased layers of neurons in CA1 region. When neurons are damaged by external abnormal stimulation, the dissolution and disappearance of Nissl body - the unique structure of neurons, can be observed (Yan et al. 2018). Our stain results showed that compared with adult group, Nissl body staining is obviously lighter in both cortex and hippocampus in aging rat.

Icariin, as a major bioactive component for both Chinese herb and formula has been found to evoke the neuroprotective effects both *in vivo* and *in vitro* (Zou et al. 2020). For instances, icariin can improve learning dysfunction, and restore synaptic and cognitive deficits in AD mice (Urano and Tohda 2010; Chen et al. 2016; Sheng et al. 2017). In addition, icariin can significantly improve dopaminergic neuronal loss and neuroinflammation in PD mice (Wang et al. 2018; Zhang et al. 2019). Furthermore, icariin ameliorates lipopolysaccharide induced brain dysfunction, as well as corticosterone-induced apoptosis in neurons. Consistent with this evidence, our data showed that icariin could obviously alleviate neuron damage of aging rat, specifically by reducing neurodegeneration in cortex and hippocampal CA3 region as well as increasing neuron number in hippocampal CA1 region. Moreover, after icariin treatment, Nissl bodies are increased both in cortex and hippocampus of aging rat.

As terminally differentiated cells, neurons maintain the specific cellular functions essentially depend on autophagy. Although excessive autophagy can lead to neuronal damage (Zhang et al. 2012; Gerónimo-Olvera et al. 2017), it is widely accepted that basal autophagy level was positive co-relation with memory function in elderly. Disruption the Beclin1-BCL2 complex was reported to enhance basal autophagy and extend lifespan (Fernández et al. 2018), which also can improve the memory dysfunction in aging drosophila (Gupta et al. 2013). The mice with Atg7 deficiency showed obvious behavioural abnormality after birth (Komatsu et al. 2006). In addition, deletion of lysosomal hydrolase inhibitor gene cystatin B effectively improve learning function via inducing autophagy in AD mice (Wang et al. 2016). Dysfunction autophagy during aging have been well proved, which is related to a variety of neurodegenerative diseases (Bergamini 2006; Nixon 2013). Our present study found that the expression of autophagy related proteins-LC3B, Beclin1 and Atg5 are significant decreased, while p62 is increased in aging rat. Immunofluorescence result showed that LC3 was mainly co-localized with neurons. ICA treatment can obviously reverse the decline in autophagy, manifested by increased LC3B, Atg5 and Beclin1 protein level, and decreased p62 accumulation. These results indicate that ICA alleviated aging related neuronal dysfunction mainly through improve basal autophagy level in aging rat.

ULK1 is a serine/threonine protein kinase that combines with Atg13, FIP200 and Atg101 to form a complex and participates in the initial stage of autophagy (Shang and Wang 2011). ULK1 can be phosphorylated at different sites via mTOR or AMPK resulting in completely different physiological responses-inhibit autophagy or activate autophagy (Kim et al. 2011). Under starvation, AMPK is activated, which in turn phosphorylates multiple sites of ULK1 and activates autophagy (Hosokawa et al. 2009). Several studies found that AMPK activation can improve energy metabolism and protein clearance in the brains of neurodegenerative disease patients (Vingtdeux et al. 2010; Ashabi et al. 2014; Bayliss et al. 2016). Activating AMPK could alleviate pathological injury in MPTP induced-dopaminergic neurotoxicity model (Lu et al. 2016). Moreover, pharmacologically activating AMPK effectively increase autophagy flux and delay brain aging in senescence accelerated mouse-8 (Xu et al. 2020). Hence, regulating AMPK activation might be a useful therapeutic target in neurodegenerative disease. In our study, compared with adult group, the expression of p-AMPK is decreased, as well as p-mTOR and p-ULK1 (S757) are increased in the aging rat. After administration with ICA, the expression of p-AMPK was improved and the activation of p-mTOR and p-ULK1 (S757) was inhibited. Based on these results, ICA improves autophagy level mainly through activation AMPK follow by inhibiting mTOR and ULK1 (S757) activation.

## Conclusions

Our data indicated that ICA can effectively improve the morphology of neurons in cortex and hippocampal of aging rats. The underlying mechanism is that ICA could enhance neuronal autophagy level through activating AMPK and inhibiting the activation of mTOR, thereby inhibiting the phosphorylation of serine at ULK1 (Ser757) site.
